# Effect of hepatic or renal impairment on the pharmacokinetics of evacetrapib

**DOI:** 10.1007/s00228-016-2017-1

**Published:** 2016-02-09

**Authors:** David S. Small, Wei Zhang, Jane Royalty, Ellen A. Cannady, Delyn Downs, Demetrio Ortega, Jeffrey G. Suico

**Affiliations:** Lilly Research Laboratories, Eli Lilly and Company, Lilly Corporate Center, Indianapolis, IN 46285 USA; Covance Inc., Evansville, IN 47710 USA

**Keywords:** Evacetrapib, Hepatic impairment, Renal impairment, Cholesteryl ester transfer protein, Pharmacokinetics

## Abstract

**Purpose:**

The aim of this study is to investigate the effect of hepatic or renal impairment on the pharmacokinetics of a single 130-mg evacetrapib dose.

**Methods:**

Two open-label, parallel-design studies in males and females with normal hepatic function or Child-Pugh mild, moderate, or severe hepatic impairment, or with normal renal function or severe renal impairment. Non-compartmental pharmacokinetic parameters were estimated from plasma concentration-time data. Evacetrapib safety and tolerability were assessed.

**Results:**

Pharmacokinetic parameter estimates were comparable between controls and mildly hepatically impaired subjects. Geometric mean area under the concentration-time curve (AUC) was greater, half-life (t_1/2_) was longer, and maximum concentration (C_max_) was lower in subjects with moderate and severe hepatic impairment than in controls. Apparent clearance (CL/F) did not differ between controls and those with mild hepatic impairment, but CL/F decreased for moderate and severe impairment. Spearman correlation coefficient showed no relationship between CL/F and Child-Pugh score. In the renal study, AUC and t_1/2_ were similar between groups, while C_max_ was 15 % lower in subjects with severe impairment. CL/F in severely renally impaired subjects differed by <6 % from that in controls. Spearman correlation coefficient showed no apparent relationship between CL/F and estimated creatinine clearance or glomerular filtration rate. Neither study noted changes in clinical laboratory parameters or clinically significant findings. Adverse event incidence was low, and all were mild or moderate in severity.

**Conclusion:**

Evacetrapib exposure did not differ between mild hepatic impairment and normal hepatic function, but increased along the progression from mild to moderate to severe hepatic impairment. Severe renal impairment did not affect evacetrapib exposure.

**Electronic supplementary material:**

The online version of this article (doi:10.1007/s00228-016-2017-1) contains supplementary material, which is available to authorized users.

## Introduction

Treatments to aggressively lower low-density lipoprotein cholesterol (LDL-C) have been shown to reduce cardiovascular events [1–3], but there remains an unmet need for additional therapies targeting other lipid-related risk factors to address residual cardiovascular disease [4]. The Framingham Study revealed that high-density lipoprotein cholesterol (HDL-C) is an important factor in cardiovascular disease, indicating that higher levels of HDL-C are associated with a lower risk of adverse cardiovascular events [5–7]. A new class of compounds inhibits cholesteryl ester transfer protein (CETP), which promotes the exchange and net transfer of triglycerides and cholesterol esters between lipoproteins, and may provide favorable benefits toward lowering cardiovascular risk [8, 9]. Evacetrapib is a potent and selective inhibitor of CETP that has demonstrated its ability to increase HDL-C and decrease LDL-C [10–13]. A human metabolism study in healthy subjects given a single oral dose of evacetrapib showed extensive hepatic metabolism of evacetrapib, identifying evacetrapib and two metabolites in plasma [14]. Most (93.1 %) of the administered dose was eliminated in the feces, while only 2.30 % of the dose was excreted in urine. In vitro data indicated that oxidative metabolism by cytochrome P450 (CYP) enzymes (predominately CYP3A and, to a lesser extent, CYP2C8) is primarily responsible for evacetrapib clearance [14]. Thus, hepatic impairment, but not renal impairment, could be expected to alter evacetrapib’s pharmacokinetic (PK) profile.

The patient population for evacetrapib anticipated treatment in those with hepatic or renal impairment, which could affect the PK of evacetrapib leading to changes in safety, tolerability, and/or efficacy of the drug. Two studies investigated the effect of hepatic and renal impairment on the PK of a single oral dose of 130-mg evacetrapib (NLM Identifier: NCT01836185 and NCT01825889) [15, 16], the dose used in evacetrapib's phase 3 study (NLM Identifier: NCT01687998) [17]. This report presents the results of the PK, safety, and tolerability after a single oral dose of 130 mg of evacetrapib in subjects with mild, moderate, and severe hepatic impairment or with severe renal impairment compared to healthy control subjects.

## Materials and methods

### Clinical study designs

All subjects received information about the study purposes and potential risks and provided written informed consent before beginning any study procedures. Protocols and informed consent forms were approved by the institutional review board (Schulman Associates Investigational Review Board, Inc., Sunrise, Florida, USA). Both studies were conducted in accordance with regulatory guidances and good clinical practice guidelines. Subjects in both studies were allowed to continue taking concomitant medications, which were withheld for 4 h after the evacetrapib dose unless the investigator felt they were necessary for safety reasons. Subjects taking strong inducers or inhibitors of CYP3A4 and CYP2C8 were excluded.

#### Hepatic impairment

This open-label, single-dose, parallel-design study evaluated evacetrapib at multiple study sites in subjects with hepatic impairment or with normal hepatic function, as classified at screening using the Child-Pugh system (NLM identifier: NCT01836185) [15, 18–20]. The study design adheres to the US Food and Drug Administration (FDA) guidance for studies in which hepatic metabolism and/or excretion accounts for >20 % of the elimination of a parent drug or active metabolite [20]. Thirty-two subjects were enrolled to ensure that at least 30 subjects completed the study: at least 8 subjects with normal hepatic function, mild impairment (Child-Pugh Class A), and moderate hepatic impairment (Child-Pugh Class B) and at least 6 subjects with severe hepatic impairment (Child-Pugh Class C). The sample size was based on FDA guidance [20], which advises that at least six subjects in each group are required to provide evaluable data, and was selected to achieve the study objectives rather than to satisfy an a priori statistical requirement.

Subjects were males and females not of childbearing potential between 42 and 77 years of age, inclusive, with a body mass index (BMI) of 19.1 to 39.8 kg/m^2^, inclusive, at the time of screening. Creatinine clearance (CLcr) was required to be ≥70 mL/min as estimated using the Cockcroft-Gault equation (Supplemental Equation 1), based on serum creatinine and screening body weight [21]. Subjects with normal hepatic function were required to be generally healthy individuals with acceptable clinical laboratory test results and vital signs. Subjects with mild to severe hepatic impairment had a diagnosis of chronic hepatic impairment for at least 6 months, with no clinically significant changes within 90 days prior to dosing. Preexisting conditions causing hepatic impairment included hepatitis C, hepatic cirrhosis, alcoholic cirrhosis, and hepatic steatosis. Stable baseline conditions were permitted in the hepatically impaired groups, provided that the condition or required treatment would not negatively impact subject health or study conduct. Subjects with normal hepatic function were matched by age (±10 years), sex, and BMI (±20 %) to subjects with mild, moderate, and severe hepatic impairment.

Subjects were admitted to the Clinical Research Unit (CRU) on the day prior to dosing and resided in the CRU for at least 48 h up to 15 days after evacetrapib administration; all subsequent assessments were conducted on an outpatient basis. Subjects were administered a single oral dose of 130-mg evacetrapib in the morning following an 8-h fast. Subjects were required to return to the CRU for a follow-up visit at least 28 days after the evacetrapib dose.

#### Renal impairment

This was a multicenter, open-label, single-dose, parallel-design study evaluating evacetrapib in male and female subjects with severe renal impairment or with normal renal function (NLM identifier: NCT01825889) [16]. The study design considered the current regulatory guidances for the study of PK in subjects with impaired renal function; however, modifications to the approach within the FDA guidance were made [22–24].

Since only 2 % of the evacetrapib dose is eliminated in the urine, renal impairment was not expected to substantially affect evacetrapib PK. Therefore, a reduced study design in only severely renally impaired subjects and healthy subjects was planned. A planned interim data review was conducted following dosing of five subjects with severe renal impairment and five subjects with normal renal function. The purpose was to analyze safety, tolerability, and PK data to determine whether the reduced design sufficed or a “full” study evaluating mild, moderate, and severe renal impairment groups would be required. Since the PK difference between subjects with normal renal function and subjects with severe renal impairment was as expected, and safety and tolerability was deemed acceptable, five more subjects were enrolled into each group and the reduced study design was completed.

Twenty subjects were enrolled in the reduced study to ensure that at least 16 subjects (8 per group) completed the study. This sample size has been shown to suffice for studies of this type; it was not selected to satisfy an a priori statistical requirement. However, eight subjects per group provided at least 90 % power to demonstrate that the 90 % confidence interval (CI) for the ratio of area under the curve (AUC) and C_max_ falls within 0.5 to 2.0 compared to healthy subjects. This calculation assumed a coefficient of variation of 41 % or less, and a true ratio of geometric means of 1 for AUC and C_max_, based on previous evacetrapib study results.

Subjects were males and females (not of childbearing potential) between 42 and 78 years of age, inclusive, with a BMI of 19.5 to 39.7 kg/m^2^, inclusive, at the time of screening. Creatinine clearance was estimated at screening using the Cockcroft-Gault equation (Supplemental Equation 1), and glomerular filtration rate was estimated using the Modification of Diet in Renal Disease (MDRD) equation (Supplemental Equation 2) as described in the FDA guidance [21, 23]. Subjects with severe renal impairment were defined by an estimated CLcr <30 mL/min, using the Cockcroft-Gault equation, but not requiring dialysis (Supplemental Table 1). Data from these subjects were compared to those from control subjects, who were matched by demographics and had normal renal function (CLcr ≥90 mL/min). Subjects with normal renal function were required to be generally healthy individuals with acceptable clinical laboratory test results and vital signs. Control subjects with normal renal function were matched for age (±10 years), sex, and BMI (±15 %) to subjects with severe renal impairment.

Subjects were admitted to the CRU on the day prior to dosing and resided in the CRU for at least 48 h postdose. All subsequent assessments were conducted on an outpatient basis. Subjects were given a single oral dose of 130-mg evacetrapib in the morning, preceded by breakfast. Subjects were required to return to the CRU for a follow-up visit at least 28 days after the evacetrapib dose.

### Pharmacokinetic assessments

#### Blood sampling

In both studies, blood samples were collected predose and 1, 2, 3, 4, 6, 8, 12, 24, 36, 48, 72, 120, 168, 216, 264, 312, and 336 h postdose for measurement of evacetrapib concentrations in plasma. In the hepatic study, blood samples were also collected at 4, 24, and 48 h postdose for assessment of plasma protein binding of evacetrapib. In the renal study, blood samples were collected prior to dosing and at 4, 24, and 72 h postdose to determine the effects of renal impairment on evacetrapib plasma protein binding.

#### Bioanalysis

Plasma samples were analyzed for evacetrapib using a validated method of liquid chromatography with tandem mass spectrometry detection at Covance Laboratories, Inc. (Madison, Wisconsin, USA; data on file). The lower limit of quantification was 1.00 ng/mL and the upper limit of quantification was 1000 ng/mL. The interassay accuracy (relative error) during validation ranged from −2.9 to 1.5 %. The interassay precision (relative standard deviation) during validation ranged from 2.9 to 6.4 %. Evacetrapib was stable in plasma for up to 365 days when stored at −20 and −70 °C.

Protein binding of evacetrapib was determined ex vivo in clinical plasma samples using an equilibrium dialysis method at Quintiles Biosciences, Inc. (Ithaca, New York, USA).

#### Pharmacokinetic analysis

Pharmacokinetic parameter estimates for evacetrapib were calculated by standard non-compartmental methods of analysis using Phoenix WinNonlin Version 6.2.1 (Pharsight Corporation, Mountain View, California, USA). Plasma concentrations below the lower limit of the assay were excluded from the analysis, except for those prior to the first measurable concentration, which were set to zero.

The PK parameters for analysis included maximum observed concentration (C_max_); time of C_max_ (t_max_); AUC of concentration versus time from zero to infinity (AUC[0-∞]); the fraction of AUC(0-∞) derived by extrapolation (%AUC[t_last_-∞]); AUC from time zero to time t_last_, where t_last_ is the last time point with a measurable concentration (AUC[0-t_last_]); apparent terminal elimination half-life (t_1/2_); apparent clearance (CL/F); and apparent volume of distribution during the terminal phase (V_Z_/F).

### Statistical analysis

Statistical analysis evaluated log-transformed AUC(0-∞), AUC(0-t_last_), and C_max_ using an analysis of variance model with group as a fixed factor. The ratio of least squares (LS) geometric means and the corresponding 90 % CI were estimated between either the renal impairment or hepatic impairment groups versus the respective control group. The analysis of t_max_ was based on a non-parametric method. Medians and differences in medians for the groups and the *p* value from a Wilcoxon rank sum test are presented. In the renal study, these analyses were conducted twice, using two different renal function group assignments: CLcr (estimated using the Cockcroft-Gault equation) and estimated glomerular filtration rate (eGFR; calculated using the MDRD equation) [23].

An analysis was also conducted using a scatterplot featuring a linear regression analysis and Spearman rank correlation coefficient [25]. In the hepatic study, a scatterplot of CL/F versus Child-Pugh score was produced including all groups on the same figure (Child-Pugh score was zero for the normal group).

In the renal study, the relationship between CL/F and both CLcr (estimated using the Cockcroft-Gault equation) and eGFR (calculated using the MDRD equation) were examined by scatterplot.

For both studies, analysis of evacetrapib protein binding included calculation of mean, standard deviation (SD), and Q-test for outliers for the fraction bound.

### Safety assessments

Safety and tolerability parameters assessed in both studies included clinical laboratory tests, vital sign measurements, physical examinations, electrocardiograms, and adverse event (AE) recording.

## Results

### Demographics and disposition

Subject demographics are presented in Table 1. In the hepatic study, 32 subjects (21 male and 11 female), age 42 to 77 years, entered the study, and all 32 completed it. Ten subjects had normal hepatic function, eight had mild impairment, eight had moderate impairment, and six had severe impairment (Supplemental Table 2). The mean age was similar across the hepatic function groups. All subjects with normal hepatic function met the prespecified matching criteria for age, sex, and BMI except for one subject whose BMI of 23.4 kg/m^2^ was 22.5 % higher than the BMI of 19.1 kg/m^2^ in his matched patient with severe hepatic impairment.

**Table 1:** Subject demographics

		Hepatic function^a^ (*N* = 32)				Renal function^b^ (*N* = 20)	
		Normal (*N* = 10)	Mild (*N* = 8)	Moderate (*N* = 8)	Severe (*N* = 6)	Normal (*N* = 10)	Severe (*N* = 10)
Age (years)	Mean	54.4	54.1	58.6	57.8	61.5	65.2
	SD	8.6	2.9	9.6	4.7	8.0	9.6
	Range	42–71	48–57	47–77	52–62	42–69	48–78
Sex	Male	6 (60 %)	4 (50 %)	6 (75 %)	5 (83 %)	6 (60 %)	6 (60 %)
	Female	4 (40 %)	4 (50 %)	2 (25 %)	1 (17 %)	4 (40 %)	4 (40 %)
Race	White	9 (90 %)	6 (75 %)	8 (100 %)	6 (100 %)	9 (90 %)	6 (60 %)
	Black or African descent	1 (10 %)	1 (12.5 %)	0 (0 %)	0 (0 %)	0 (0 %)	4 (40 %)
	Asian	0 (0 %)	1 (12.5 %)	0 (0 %)	0 (0 %)	0 (0 %)	0 (0 %)
	Multiple	0 (0 %)	0 (0 %)	0 (0 %)	0 (0 %)	1 (10 %)	0 (0 %)
Weight (kg)	Mean	85.1	78.9	93.1	79.1	84.2	79.0
	SD	14.8	14.8	14.1	17.2	12.5	19.8
	Range	62.7–115.5	61.4–108.3	80.0–115.0	50.4–98.5	64.5–103.3	55.4–121.0
BMI (kg/m^2^)	Mean	28.8	27.3	31.4	27.3	28.9	28.5
	SD	4.35	4.37	5.14	5.14	3.89	5.88
	Range	23.4–38.6	22.7–34.5	24.0–39.8	19.1–32.2	22.2–34.5	19.5–39.7
Child-Pugh Score (points) ^c^	Mean	–	5.4	7.5	10.7	–	–
	SD	–	0.5	0.5	0.8	–	–
	Range	–	5–6	7–8	10–12	–	–
CLcr (mL/min)	Mean	–	–	–	–	119.8	20.6
	SD	–	–	–	–	28.2	4.71
	Range	–	–	–	–	91.5–160.9	12.8–27.1

*BMI* body mass index, *CLcr* creatinine clearance, *N* number of subjects, *SD* standard deviation

^a^Hepatic function classified by Child-Pugh score

^b^Renal function classified by creatinine clearance estimated using the Cockcroft-Gault equation

^c^Child-Pugh Class A (mild): 5 or 6 points, Child-Pugh Class B (moderate): 7 to 9 points, Child-Pugh Class C (severe): 10 to 15 points

In the renal study, 20 subjects (12 male and 8 female), aged 42 to 78 years, inclusive, entered the study, and all 20 completed it. The Cockcroft-Gault calculation for estimating CLcr was used to assign subjects into groups for the demographics analyses. Ten of the 20 subjects had severe renal impairment and 10 had normal renal function. All subjects with normal renal function met the prespecified matching criteria for age, sex, and BMI except for one subject whose BMI of 28.5 kg/m^2^ was 17.3 % higher than the BMI of 24.3 kg/m^2^ in her matched patient with severe renal impairment.

### Pharmacokinetics

#### Hepatic impairment

**Fig 1:**
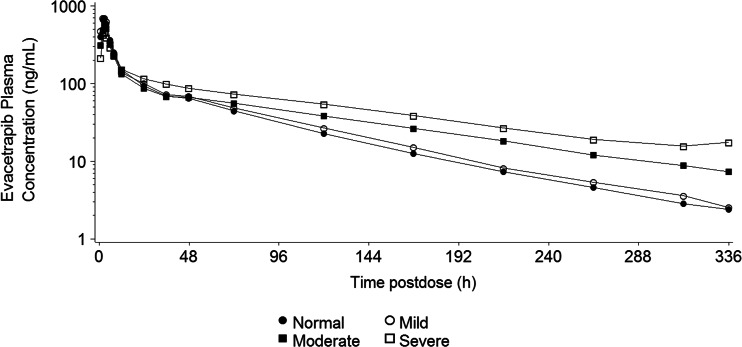
Arithmetic mean plasma concentration-time profiles of evacetrapib following a single dose of 130-mg evacetrapib in subjects with mild, moderate, and severe hepatic impairment and control subjects with normal hepatic function

The PK profiles of evacetrapib following a single 130-mg dose to subjects with hepatic impairment and control subjects with normal hepatic function are shown in Fig. 1. Estimates of AUC, C_max_, and t_1/2_ were comparable between the control subjects and subjects with mild hepatic impairment (Table 2). The geometric mean AUC was greater, t_1/2_ was longer, and C_max_ was lower in subjects with severe hepatic impairment than in subjects with normal hepatic function or mild impairment. Comparisons of parameter estimates in subjects with moderate hepatic impairment to those in patients with other degrees of impairment yielded mixed results: The C_max_ was similar to that in mildly impaired subjects, the AUC fell between that in mildly and severely impaired subjects, and the t_1/2_ was similar to that in severely impaired subjects. There was no apparent trend in t_max_ with increasing hepatic impairment; the 90 % CIs of the geometric LS means of the test (hepatic impairment group) to the reference (normal hepatic function) span unity (Table 3), and the between-subject variability in exposure within each group was high with geometric coefficients of variation of 49 to 84 % for AUC(0-t_last_), 50 to 84 % for AUC(0-∞), and 51 to 144 % for C_max_.

**Table 2:** Evacetrapib pharmacokinetic parameter estimates in subjects with normal or impaired hepatic or renal function

	Geometric Mean (CV%)					
	Hepatic function^a^				Renal function^b^	
	Normal (*N* = 10)	Mild (*N* = 8)	Moderate (*N* = 8)	Severe (*N* = 6)	Normal (*N* = 10)	Severe (*N* = 10)
AUC(0-t_last_) (ng/h/mL)	10,700 (50)	10,500 (84)	13,200 (49)	15,800 (62)	16,000 (37)	15,200 (49)
AUC(0-∞) (ng/h/mL)	10,900 (50)	10,600 (84)	13,900 (54)	17,000 (71)	16,300 (39)	15,500 (50)
%AUC(t_last_-∞) (%)	1.31 (53)	1.27 (67)	3.88 (111)	3.85 (214)	1.48 (73)	1.49 (93)
C_max_ (ng/mL)	605 (98)	609 (144)	591 (58)	478 (51)	1140 (44)	969 (43)
t_max_^c^ (h)	3.00 (2.00–6.00)	3.00 (1.00–4.00)	3.00 (2.00–4.00)	3.00 (2.00–6.00)	4.00 (2.00–4.03)	4.00 (3.00–6.00)
t_1/2_^d^ (h)	47.5 (31.5–72.7)	49.8 (33.8–68.2)	79.5 (55.6–133)	75.3 (43.9–129)	58.5 (41.7–98.6)	55.7 (35.6–89.5)
CL/F (L/h)	12.0 (50)	12.2 (84)	9.33 (54)	7.63 (71)	7.97 (39)	8.40 (50)
V_Z_/F (L)	821 (52)	878 (80)	1070 (28)	828 (20)	672 (25)	675 (45)

*AUC(0-∞)* area under the concentration-time curve from time zero to infinity, *AUC(0-t*_*last*_ *)* area under the concentration-time curve from time zero to time t_last_, where t_last_ is the last time point with a measurable concentration, *%AUC(t*_*last*_ *-∞)* percent of AUC(0-∞) derived by extrapolation, *CL/F* apparent clearance, *C*_*max*_ maximum observed drug concentration, *CV* coefficient of variation, *N* number of subjects, *t*_*1/2*_ apparent terminal elimination half-life, *t*_*max*_ time of maximum observed drug concentration, *V*_*Z*_ */F* apparent volume of distribution during the terminal phase

^a^Hepatic function classified by Child-Pugh score

^b^Renal function classified by creatinine clearance estimated using the Cockcroft-Gault equation

^c^Median (range)

^d^Geometric mean (range)

**Table 3:** Statistical analysis of the pharmacokinetic parameter estimates of evacetrapib following a single dose of 130-mg evacetrapib in subjects with normal or impaired hepatic function

Parameter	Hepatic impairment^a^	Number of subjects	Geometric LS mean	Ratio of geometric LS means test: reference (90 % CI)	
AUC(0-t_last_) (ng·h/mL)	Normal (reference)	10	10,699		
	Mild (test)	8	10,482	0.980 (0.622, 1.54)	
	Moderate (test)	8	13,154	1.23 (0.780, 1.94)	
	Severe (test)	6	15,828	1.48 (0.902, 2.43)	
AUC(0-∞) (ng·h/mL)	Normal (reference)	10	10,857		
	Mild (test)	8	10,639	0.980 (0.612, 1.57)	
	Moderate (test)	8	13,940	1.28 (0.801, 2.06)	
	Severe (test)	6	17,044	1.57 (0.940, 2.62)	
C_max_ (ng/mL)	Normal (reference)	10	605		
	Mild (test)	8	609	1.01 (0.535, 1.89)	
	Moderate (test)	8	591	0.977 (0.520, 1.84)	
	Severe (test)	6	478	0.789 (0.397, 1.57)	
Parameter	Hepatic impairment^a^	*N*	Median	Median difference test - reference(90 % CI)	*P* value
t_max_ (h)	Normal (reference)	10	3		
	Mild (test)	8	3	0.00 (−1.00, 1.00)	0.817
	Moderate (test)	8	3	0.00 (−1.00, 1.00)	0.962
	Severe (test)	6	3	0.00 (−1.00, 1.00)	0.818

*AUC(0-∞)* area under the concentration-time curve from time zero to infinity, *AUC(0-t*_*last*_ *)* area under the concentration-time curve from time zero to time t_last_, where t_last_ is the last time point with a measurable concentration, *CI* confidence interval, *C*_*max*_ maximum observed drug concentration, *LS* least squares, *N* number of subjects, *t*_*max*_ time of maximum observed drug concentration

^a^Hepatic function classified by Child-Pugh score

**Fig 2:**
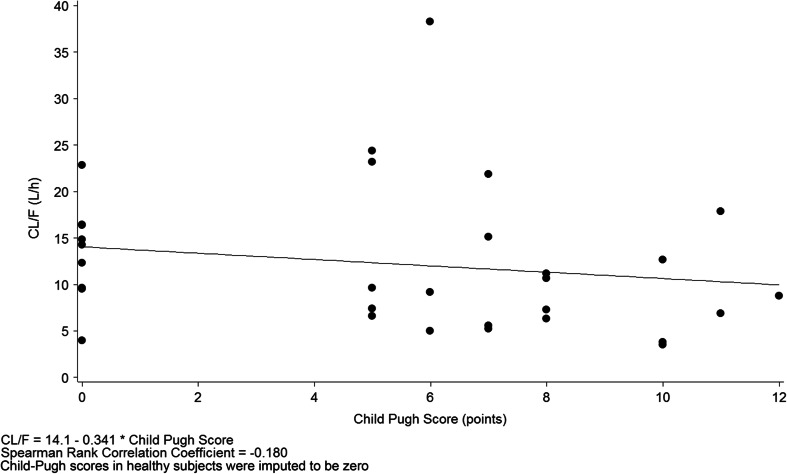
Relationship between evacetrapib apparent clearance (CL/F) and Child-Pugh score

There was no notable difference in mean CL/F between subjects with normal hepatic function and those with mild hepatic impairment, but mean CL/F was lower for the moderate and severe hepatic impairment groups than the control group (Table 2). A linear regression analysis did not show a correlation between CL/F and Child-Pugh score (Spearman rank correlation coefficient = −0.180; Fig. 2).

Evacetrapib was 97.6 ± 2.2 % (mean ± SD) bound in plasma from healthy controls and 97.4 ± 1.1, 96.9 ± 2.8, and 94.8 ± 2.1 % bound in plasma from subjects with mild, moderate, and severe hepatic impairment, respectively.

#### Renal impairment

**Fig 3:**
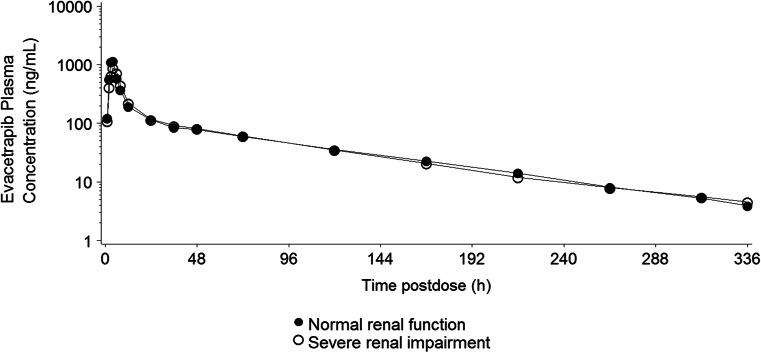
Arithmetic mean plasma concentration-time profiles of evacetrapib following a single dose of 130-mg evacetrapib in subjects with severe renal impairment and control subjects with normal renal function

The PK profiles of evacetrapib following a single 130-mg dose to subjects with renal impairment and control subjects with normal renal function are shown in Fig. 3. Although C_max_ was 15 % lower in subjects with severe renal impairment, the AUC was similar to that in subjects with normal renal function (Table 4). No apparent difference in median t_max_ was noted, and t_1/2_ was similar between the two groups (Table 2). Estimates of geometric mean CL/F and V_Z_/F in the severe renal impairment subjects differed by <6 % from those in normal subjects (Table 2). The overall results were also similar for the statistical comparisons of PK parameter estimates between the severe renal impairment group and control group when renal function was classified by eGFR calculated using the MDRD equation (Table 4).

**Table 4:** Statistical analysis of the pharmacokinetic parameter estimates of evacetrapib following a single dose of 130-mg evacetrapib in subjects with normal or severely impaired renal function

		CLcr estimated using the C-G equation			eGFR calculated using the MDRD equation		
Parameter	Renal impairment	*N*	Geometric LS mean	Ratio of geometric LS means severe: normal (90 % CI)	*N*	Geometric LS mean	Ratio of geometric LS means severe: normal (90 % CI)
AUC(0-t_last_) (ng·h/mL)	Normal	10	16,008	0.948 (0.686, 1.31)	7^a^	17,635	0.808 (0.555, 1.18)
	Severe	10	15,176		9^b^	14,246	
AUC(0-∞) (ng·h/mL)	Normal	10	16,320	0.948 (0.683, 1.32)	7^a^	18,035	0.805 (0.550, 1.18)
	Severe	10	15,478		9^b^	14,525	
C_max_ (ng/mL)	Normal	10	1142	0.849 (0.615, 1.17)	7^a^	1231	0.754 (0.509, 1.12)
	Severe	10	969		9^b^	929	

*AUC(0-∞)* area under the concentration-time curve from time zero to infinity, *AUC(0-t*_*last*_ *)* area under the concentration-time curve from time zero to time t_last_, where t_last_ is the last time point with a measurable concentration, *C-G* Cockcroft-Gault, *CI* confidence interval, *CLcr* creatinine clearance, *C*_*max*_ maximum observed drug concentration, *eGFR* estimated glomerular filtration rate, *LS* least squares, *MDRD* Modification of Diet in Renal Disease, *N* number of subjects

^a^Three subjects were excluded from the normal group when the MDRD equation was used to estimate GFR. See Discussion section for details

^b^One subject was excluded from the severe group MDRD equation was used to estimate GFR. See Discussion section for details

**Fig 4:**
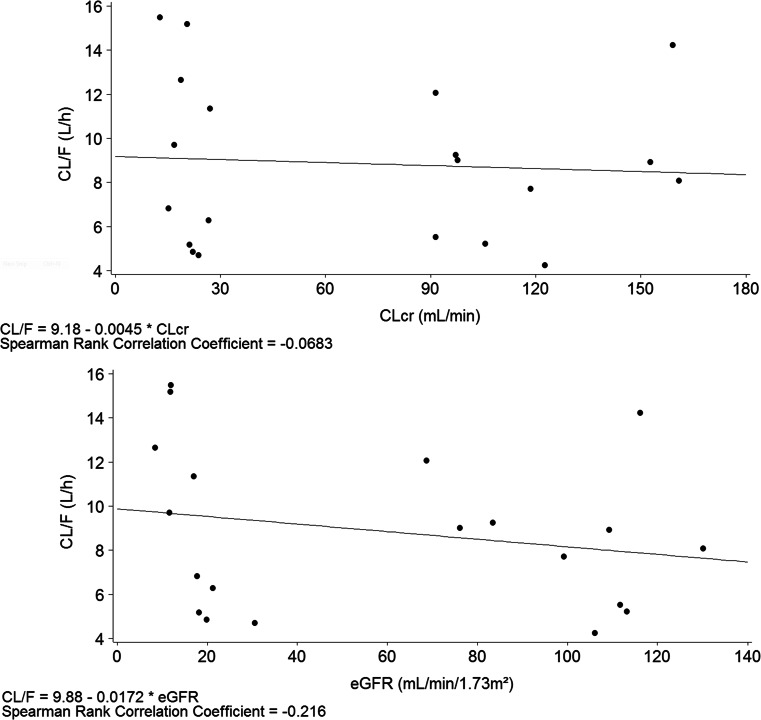
Relationship between evacetrapib apparent clearance (CL/F) and creatinine clearance (CLcr) estimated by Cockcroft-Gault equation (*upper panel*) and glomerular filtration rate (eGFR) estimated using the Modification of Diet in Renal Disease (MDRD) equation (*lower panel*)

The relationships between evacetrapib CL/F and CLcr calculated by the Cockcroft-Gault equation were examined using scatterplots featuring a linear regression analysis and Spearman rank correlation coefficient. The linear regression analysis did not show an apparent correlation between evacetrapib CL/F and CLcr (Spearman rank correlation coefficient = −0.0683; Fig. 4). Similarly, with a Spearman Rank correlation coefficient of −0.216, there was no relationship between evacetrapib CL/F and eGFR (Fig. 4).

Evacetrapib was 98.9 ± 1.0 % (mean ± SD) bound in plasma from healthy control subjects and 97.8 ± 1.7 % bound in plasma from subjects with severe renal impairment.

### Safety and tolerability

A single dose of 130-mg evacetrapib was well-tolerated by subjects with hepatic and renal impairment. All subjects completed both studies. The incidence of treatment-emergent AEs (TEAEs) was generally low across all groups, and all TEAEs were mild or moderate in severity. No notable changes in clinical laboratory parameters or clinically significant findings were noted as TEAEs in either study.

In the hepatic impairment study, 12 of the 32 subjects who received evacetrapib reported a total of 22 TEAEs, with 8 of these subjects reporting a total of 10 TEAEs considered by the investigator to be related to study drug. Of the ten study drug-related TEAEs, two were reported by subjects with normal hepatic function, two by subjects with mild impairment, two by subjects with moderate impairment, and four by subjects with severe hepatic impairment. The most common evacetrapib-related TEAEs were diarrhea, flatulence, and headache, each of which was reported by two subjects.

In the renal impairment study, 5 of the 20 subjects who received evacetrapib reported a total of 8 TEAEs, with 3 of these subjects reporting a total of 4 TEAEs considered by the investigator to be related to study drug. The four evacetrapib-related TEAEs were abdominal distension, diarrhea, muscle spasms, and dizziness.

## Discussion

Evacetrapib is cleared primarily through CYP-mediated hepatic metabolism, with minor elimination through renal excretion [14]. As the intended patient population for CETP inhibitors may include patients with hepatic and renal impairment, it was important to ascertain whether evacetrapib could be safely prescribed to these populations without dose adjustment. Two studies were conducted to determine the effect of hepatic and renal impairment on the PK of evacetrapib.

The hepatic impairment study evaluated evacetrapib PK in subjects with impaired hepatic function classified by Child-Pugh scores as mild, moderate, and severe impairment. Since the liver is involved in evacetrapib’s metabolism, we hypothesized that hepatic impairment categorized by Child-Pugh scores might increase evacetrapib exposure by reducing evacetrapib clearance. The Child-Pugh scale is commonly used to classify subjects with hepatic impairment in clinical trials; it was originally developed to predict mortality during surgery [19] and is now used to assess the status of chronic liver disease and cirrhosis. Evacetrapib exposure increased with increasing degree of hepatic impairment determined by Child-Pugh score, with an increase in AUC(0-t_last_) in the moderate and severe hepatic impairment groups of 23 and 48 %, respectively, relative to the normal hepatic function group. The physiological basis for this pattern could be reduced clearance due to impaired perfusion and/or impaired metabolism.

The renal impairment study examined evacetrapib PK in subjects with severe renal impairment compared to subjects with normal renal function. Renal impairment did not appreciably affect evacetrapib exposure, consistent with previous data showing that only about 2 % of a radiolabeled evacetrapib dose was excreted in the urine of healthy subjects [14]. Typically, if renal clearance contributes significantly to the overall clearance, then as renal function worsens, one would expect AUC to increase. With evacetrapib, the PK was not significantly altered; thus, no dose adjustment based solely on exposure would be expected for patients with impaired renal function. These results can be extrapolated to suggest that evacetrapib absorption and non-renal routes of evacetrapib disposition, such as metabolism by CYP3A and CYP2C8, are not substantially affected by severe renal impairment.

There was no apparent relationship between evacetrapib CL/F and CLcr. The classification of CLcr estimated by the Cockcroft-Gault equation and the classification of eGFR estimated by the MDRD equation resulted in similar trends, although differences in the specific ratios of geometric least square means between the renal function groups were observed between the CLcr and eGFR methods (Table 4). The differences resulted from four subjects (three from the normal group; one from the severe renal impairment group) who were included in the analysis by CLcr but not the analysis by eGFR because they fell outside the classification boundaries for normal renal function (CLcr ≥90 mL/min or eGFR ≥90 mL/min/1.73 m^2^) and severe renal impairment (CLcr <30 mL/min or eGFR <30 mL/min/1.73 m^2^) when the MDRD equation was used to estimate GFR. The three subjects excluded from the normal group had AUC estimates at the lower end of that group, whereas the one subject excluded from the severely impaired group had the highest exposure in that group. Removing these subjects’ data from the eGFR statistical analysis had the effect of increasing the geometric least square means for AUC(0-t_last_) and AUC(0-∞) in the normal group and decreasing the estimates of these parameters in the severely impaired group, thus widening the gap between estimates in the two groups.

A difference in exposure is apparent between the control subjects in the renal and hepatic impairment studies, with mean AUC(0-∞) of 16,300 ng·h/mL and 10,900 ng·hr/mL, respectively. A high-fat, high-calorie meal increases AUC by 44 % on average [26]. Hepatic study subjects were fasted, but renal study subjects had breakfast before dosing. The food effect, combined with potential study-to-study variability, would appear to explain most or all of this difference.

A single dose of evacetrapib was well-tolerated by subjects with hepatic and renal impairment and normal control subjects. There were no notable differences in safety profiles, incidence of TEAEs was low across all groups, and all TEAEs were mild or moderate in severity. While the incidence of TEAEs was numerically higher in the hepatic impairment groups than in subjects with normal hepatic function, there did not appear to be a correlation between drug exposure and incidence of TEAEs with TEAE onset frequently occurring after at least one half-life.

In summary, a single oral dose of 130 mg evacetrapib showed no discernible difference in exposure between mild hepatic impairment and normal hepatic function, although exposure increased along the progression from mild to moderate to severe hepatic impairment. Severe renal impairment did not affect evacetrapib exposure.

## Electronic supplementary material

ESM 1 (DOCX 11.5 kb)

ESM 2 (DOCX 11.5 kb)

Supplemental Table 1 (DOCX 12.1 kb)

Supplemental Table 2 (DOCX 13.1 kb)

## References

[1] Barter P, Gotto AM, LaRosa JC, Maroni J, Szarek M, Grundy SM, Kastelein JJ, Bittner V, Fruchart JC, Treating to New Targets Investigators (2007). HDL cholesterol, very low levels of LDL cholesterol, and cardiovascular events. N Engl J Med.

[2] Di Angelantonio E, Sarwar N, Perry P, Kaptoge S, KK R, Thompson A, AM W, Lewington S, Sattar N, CJ P, Collins R, SG T, Danesh J, Emerging Risk Factors Collaboration (2009). Major lipids, apolipoproteins, and risk of vascular disease. Jama.

[3] Baigent C, Blackwell L, Emberson J, LE H, Reith C, Bhala N, Peto R, EH B, Keech A, Simes J, Collins R, Cholesterol Treatment Trialists’ Collaboration (2010). Efficacy and safety of more intensive lowering of LDL cholesterol: a meta-analysis of data from 170,000 participants in 26 randomised trials. Lancet.

[4] Bays H, Stein EA (2003). Pharmacotherapy for dyslipidaemia—current therapies and future agents. Expert Opin Pharmacother.

[5] Gordon T, Castelli WP, Hjortland MC, Kannel WB, Dawber TR (1977). High density lipoprotein as a protective factor against coronary heart disease. The Framingham Study. Am J Med.

[6] Castelli WP, Garrison RJ, Wilson PW, Abbott RD, Kalousdian S, Kannel WB (1986). Incidence of coronary heart disease and lipoprotein cholesterol levels. The Framingham study. Jama.

[7] Wilson PW, Abbott RD, Castelli WP (1988). High density lipoprotein cholesterol and mortality. Framingham Heart Study Arterioscler.

[8] Tall AR (1993). Plasma cholesteryl ester transfer protein. J Lipid Res.

[9] Hewing B, Fisher EA (2012). Rationale for cholesteryl ester transfer protein inhibition. Curr Opin Lipidol.

[10] Cao G, Beyer TP, Zhang Y, Schmidt RJ, Chen YQ, Cockerham SL, Zimmerman KM, Karathanasis SK, Cannady EA, Fields T, Mantlo NB (2011). Evacetrapib is a novel, potent, and selective inhibitor of cholesteryl ester transfer protein that elevates HDL cholesterol without inducing aldosterone or increasing blood pressure. J Lipid Res.

[11] Nicholls SJ, Brewer HB, Kastelein JJ, Krueger KA, Wang MD, Shao M, Hu B, McErlean E, Nissen SE (2011). Effects of the CETP inhibitor evacetrapib administered as monotherapy or in combination with statins on HDL and LDL cholesterol: a randomized controlled trial. Jama.

[12] Suico JG, Wang MD, Friedrich S, Cannady EA, Konkoy CS, Ruotolo G, Krueger KA (2014). Effects of the cholesteryl ester transfer protein inhibitor evacetrapib on lipoproteins, apolipoproteins and 24-hour ambulatory blood pressure in healthy adults. J Pharm Pharmacol.

[13] Teramoto T, Takeuchi M, Morisaki Y, Ruotolo G, Krueger KA (2014). Efficacy, safety, tolerability, and pharmacokinetic profile of evacetrapib administered as monotherapy or in combination with atorvastatin in Japanese patients with dyslipidemia. Am J Cardiol.

[14] Cannady EA, Suico JG, Wang M, Rehmel JLF, Yi P, Small DS, Zhang W, Krueger KA (2015). Evacetrapib: in vitro and clinical disposition, metabolism, excretion, and assessment of drug interactions with strong CYP3A and CYP2C8 inhibitors. Pharmacol Res Perspect.

[15] Eli Lilly and Company Pharmacokinetics of evacetrapib (LY2484595) in subjects with hepatic impairment. In: ClinicalTrials.gov [Internet]. Bethesda (MD): National Library of Medicine (US). [cited 2014 Aug 18]. Available from: http://clinicaltrials.gov/show/ NCT01836185. NLM Identifier: NCT01836185

[16] Eli Lilly and Company Pharmacokinetics of evacetrapib (LY2484595) following administration to subjects with impaired renal function. In: ClinicalTrials.gov [Internet]. Bethesda (MD): National Library of Medicine (US). [cited 2014 Aug 18]. Available from: http://clinicaltrials.gov/show/ NCT01825889. NLM Identifier: NCT01825889

[17] Eli Lilly and Company ACCELERATE. Assessment of clinical effects of cholesteryl ester transfer protein inhibition with evacetrapib in patients at a high-risk for vascular outcomes. In: ClinicalTrials.gov [Internet]. Bethesda (MD): National Library of Medicine (US). [cited 2014 Aug 18]. Available from: http://clinicaltrials.gov/show/NCT01687998 NLM. Identifier: NCT01687998

[18] Child CG, Turcotte JG (1964). Surgery and portal hypertension. Major Probl Clin Surg.

[19] Pugh RN, Murray-Lyon IM, Dawson JL, Pietroni MC, Williams R (1973). Transection of the oesophagus for bleeding oesophageal varices. Br J Surg.

[20] US Department of Health and Human Services, Food and Drug Administration, Center for Drug Evaluation and Research, Center for Biologics Evaluation and Research (2003) Guidance for Industry: Pharmacokinetics in Patients with Impaired Hepatic Function: Study Design, Data Analysis, and Impact on Dosing and Labeling Available from: http://www.fda.gov/downloads/Drugs/GuidanceComplianceRegulatoryInformation/Guidances/ucm072123.pdf. Accessed September 3, 2014.

[21] Cockcroft DW, Gault MH (1976). Prediction of creatinine clearance from serum creatinine. Nephron.

[22] European Medicines Agency Committee for Medicinal Products for Human Use (2004) Note for Guidance on the Evaluation of the Pharmacokinetics of Medicinal Products in Patients with Impaired Renal Function Available from: http://www.ema.europa.eu/docs/en_GB/document_library/Scientific_guideline/2009/09/WC500003123.pdf. Accessed August 29, 2014

[23] US Department of Health and Human Services, Food and Drug Administration, Center for Drug Evaluation and Research (2010) Guidance for Industry: Pharmacokinetics in Patients with Impaired Renal Function - Study Design, Data Analysis, and Impact on Dosing and Labeling Available from: http://www.fda.gov/downloads/Drugs/GuidanceComplianceRegulatoryInformation/Guidances/UCM204959.pdf. Accessed August 22, 2014

[24] Zhang L, Xu N, Xiao S, Arya V, Zhao P, Lesko LJ, Huang SM (2012). Regulatory perspectives on designing pharmacokinetic studies and optimizing labeling recommendations for patients with chronic kidney disease. J Clin Pharmacol.

[25] Armitage P, Berry G (1994). Statistical methods in medical research.

[26] Small DS, Zhang W, Royalty J, Cannady EA, Downs D, Friedrich S, Suico JG (2015). A multidose study to examine the effect of food on evacetrapib exposure at steady state. J Cardiovasc Pharmacol Ther.

