# Supplementary material for: Effect of hepatic or renal impairment on the pharmacokinetics of evacetrapib
Source: Eur J Clin Pharmacol. 2016 Feb 9;72:563–72. doi: 10.1007/s00228-016-2017-1 (PMC4834099; doi:10.1007/s00228-016-2017-1)
Supplement: Supplementary file 1 — (DOCX 11.5 kb) [file 228_2016_2017_MOESM1_ESM.docx]

**Supplemental Information**

**Supplemental Equation 1** Cockcroft-Gault equation for calculation of creatinine clearance [23]

If creatinine was measured in mg/100 mL:

**Male:** $\frac{\left( 140-age \right) \times(weight in kg)}{72 \times(serum creatinine in mg/100mL\mathbf{)}}$

**Female:** $\frac{\left( 140-age \right) \times\left( weight in kg \right) \times0.85}{72 \times(serum creatinine in mg/100mL)}$

If creatinine was measured in mol/L, replace 72 with 0.81.
