# Supplementary material for: Effect of hepatic or renal impairment on the pharmacokinetics of evacetrapib
Source: Eur J Clin Pharmacol. 2016 Feb 9;72:563–72. doi: 10.1007/s00228-016-2017-1 (PMC4834099; doi:10.1007/s00228-016-2017-1)
Supplement: Supplementary file 2 — (DOCX 11.5 kb) [file 228_2016_2017_MOESM2_ESM.docx]

**Supplemental Equation 2** Modification of Diet in Renal Disease abbreviated equation for calculation of estimated glomerular filtration rate [23]

eGFR (mL/min/1.73^2^) =

$$175\times{(serum creatinine)}^{-1.154}\times({age)}^{-0.203}\times\left( 0.742 if female \right)\times(1.212 if African descent)$$
