# Supplementary material for: Effect of hepatic or renal impairment on the pharmacokinetics of evacetrapib
Source: Eur J Clin Pharmacol. 2016 Feb 9;72:563–72. doi: 10.1007/s00228-016-2017-1 (PMC4834099; doi:10.1007/s00228-016-2017-1)
Supplement: Supplementary file 3 — (DOCX 12.1 kb) [file 228_2016_2017_MOESM3_ESM.docx]

**Supplemental Table 1** Renal Function Classifications for Primary Analyses

| **Group** | **Description** | **CLcr** **(mL/min)a** | **eGFR (mL/min/1.73 m2)b** |
| --- | --- | --- | --- |
| 1 | Normal renal function (control) | ≥90 | ≥90 |
| 2 | Severe renal impairmentc | <30 | <30 |

Abbreviations: CLcr = creatinine clearance; eGFR = estimated glomerular filtration rate; MDRD = Modification of Diet in Renal Disease.

^a^ Per enrollment classification; CLcr calculated using the Cockcroft‑Gault equation.

^b^ Estimated glomerular filtration rate calculated using the MDRD equation.

^c^ Not on dialysis.
