# Supplementary material for: Effect of hepatic or renal impairment on the pharmacokinetics of evacetrapib
Source: Eur J Clin Pharmacol. 2016 Feb 9;72:563–72. doi: 10.1007/s00228-016-2017-1 (PMC4834099; doi:10.1007/s00228-016-2017-1)
Supplement: Supplementary file 4 — (DOCX 13.1 kb) [file 228_2016_2017_MOESM4_ESM.docx]

**Supplemental Table 2** Baseline Parameters used to Calculate Child-Pugh Score for Classification of Hepatic Function in Subjects with Impaired Hepatic Function

| **Parameter** | **Normal a N=10** | **Mild N=8** | **Moderate N=8** | **Severe N=6** |
| --- | --- | --- | --- | --- |
| Serum Albumin (g/dL) b | 4.23 (0.35) | 4.19 (0.36) | 3.93 (0.43) | 2.78 (0.44) |
| Total Serum Bilirubin (mg/dL) b | 0.497 (0.12) | 0.561 (0.29) | 1.00 (0.58) | 2.47 (1.08) |
| Prothrombin Time INR b |  | 1.0 (0.04) | 1.1 (0.12) | 1.5 (0.41) |
| Ascites c |  | Absent (7) Slight (1) Moderate (0) | Absent (0) Slight (4) Moderate (4) | Absent (0) Slight (0) Moderate (6) |
| Encephalopathy c, d |  | Grade 0 (6) Grade 1 or 2 (2) Grade 3 or 4 (0) | Grade 0 (1) Grade 1 or 2 (7) Grade 3 or 4 (0) | Grade 0 (0) Grade 1 or 2 (5) Grade 3 or 4 (1) |

Abbreviations: INR = international normalized ratio.

^a^ Child-Pugh scores were not calculated for normal subjects. Baseline serum albumin and total serum bilirubin values are provided for reference.

^b^ Mean (standard deviation).

^c^ Severity (number of subjects).

^d^ Grade 0: normal consciousness, personality, neurological examination, electroencephalogram.

Grade 1: restless, sleep disturbed, irritable/agitated, tremor, impaired handwriting, 5 cycles per second waves.

Grade 2: lethargic, time-disoriented, inappropriate, asterixis, ataxia, slow triphasic waves.

Grade 3: somnolent, stuporous, place-disoriented, hyperactive reflexes, rigidity, slower waves.

Grade 4: unarousable coma, no personality/behavior, decerebrate, slow 2 to 3 cycles per second delta activity.
